# Impacts of Schmallenberg virus infection in early lambing sheep flocks following the second wave of virus circulation in South West England in 2012/2013: a mixed-methods descriptive study

**DOI:** 10.18849/ve.v8i1.604

**Published:** 2023-02-08

**Authors:** Michael Glover+, Neil Blake+, Clare J Phythian+

**Keywords:** SCHMALLENBERG VIRUS, MIXED-METHODS, IMPACTS, EARLY LAMBING, MALFORMATIONS, WELFARE, REPRODUCTIVE PERFORMANCE

## Abstract

**Background:**

The first cases of Schmallenberg virus (SBV) infection in the UK were confirmed in congenitally malformed lambs born in South East England in January 2012. Epidemiological studies confirmed that SBV infection could have severe negative impacts on animal welfare and productivity in affected flocks of sheep (*Ovis aries*), but there was a lack of specific research on the impacts of infection on recorded reproductive performance, animal welfare, financial performance, and farmers’ emotional well-being in some of the first affected early lambing flocks in South West England in 2012 / 2013.

**Objectives:**

This veterinary practice-based study aimed to describe the clinical signs observed by sheep farmers in the region experiencing outbreaks of disease due to SBV for the first time; to evaluate physical records (quantitative data) on reproductive performance in early lambing flocks prior to and during the affected 2012 / 2013 production year; and to gauge farmers’ perceptions (qualitative interview data and quantitative severity scores) of the impacts of SBV infection on animal welfare, financial performance, and their emotional well-being, and risks of future outbreaks of disease and preventive strategies including SBV vaccination.

**Evidentiary value:**

This mixed-methods descriptive study reported farmers’ detailed experiences, and recorded and perceived impacts, of SBV infection in six pedigree and purebred flocks in South West England, lambing early between November 2012 and January 2013. Previous surveys were larger than the current study and assessed the impacts of SBV at farm rather than flock level and on a more diverse range of British sheep farms lambing over extended periods; impacts were compared between three categories of farms based on laboratory confirmation or farmers’ suspicions of infection i.e. ‘SBV confirmed’, ‘SBV suspected’ and ‘SBV not suspected’. This study was able to capture and compare the reproductive performance of these flocks in the SBV affected production year in 2012 / 2013 with up to three previous unaffected years; it found variable negative effects of SBV not only on ewe and lambs losses, stillbirths and abortions, levels of dystocia and need for variable levels of assistance to deliver lambs, but also on overall flock reproductive performance, barren rate, lambing percentage and lamb rearing percentage. The qualitative elements of the study enabled new insights into the variable effects of SBV infection in flocks on ewes and lambs and on farmers’ perceptions of the impacts on animal welfare, flock financial performance and their own emotional well-being. The findings suggest previous surveys may not have fully captured the impacts of SBV infection in high value pedigree and purebred early lambing flocks infected for the first time during the second wave of virus circulation and peak midge vector activity in the southwest region in summer and autumn 2012. The findings highlight the need for further research to explore factors influencing uptake of SBV vaccination by farmers to protect flocks against future waves of infection, and to develop more rational vaccination programmes based on ‘early warning’ surveillance systems.

**Methods:**

Face-to-face semi-structured interviews were conducted in 2013. Qualitative interview data were thematically analysed to gain an understanding of the perspectives, perceptions and lived experiences of sheep farmers. Quantitative data in the form of (i) farmers’ self-appraised severity scores of the perceived impacts on flock welfare, financial performance and their emotional well-being; and (ii) flock records of pregnancy scanning results, lambing dates, and mortality records for ewes and lambs, were collected for the SBV affected 2012/2013 production year and for up to 3 previous years for comparison.

**Results:**

Farmers perceived generally high but widely variable negative impacts of SBV infection on animal welfare (median score: 3.5/5, range: 2–5), financial performance (median score: 3.5/5, range: 2–5) and their own well-being (median score: 4/5, range: 2–5); variation between farmers in the severity of impacts appeared not to be directly related to recorded lamb losses (of all lambs born, an overall average of 21% (range: 13.7–42.6%) were stillborn or died within 7 days; 15% (range: 4.1–42.6%) were stillborn or died due to SBV), or to reductions in lamb rearing percentage (10–37% fewer lambs reared in the affected year compared to previous reproductive performance or an industry benchmark). The qualitative elements of the study enabled new insights into the variable effects of SBV infection in flocks on ewes and lambs and on farmers’ perceptions of the impacts on animal welfare, flock financial performance and their own emotional well-being. The semi-structure interviews captured narrative descriptions of the distressing clinical signs seen in ewes and lambs, the variable levels of dystocia, and the lived experiences of farmers caring for affected sheep including the increased workload during the lambing period, greater feelings of tiredness and anxiety than in ‘normal’ lambing periods, depression, and also more positive emotions of resilience and ability to cope with an unexpected and novel disease outbreak. Three of the six farmers subsequently vaccinated with SBV vaccine to protect their early lambing flocks before the next early breeding season. Of the three farmers who decided not to vaccinate: one delayed the start of the subsequent breeding season; the second felt uncertain about using the rapidly developed and authorised vaccine so close to the start of the breeding season but was reassured by veterinary advice that the risk of a further disease outbreak in the subsequent breeding season was low as flock SBV seroprevalence was high (~90% of ewe were seropositive) following the first outbreak; and the third experienced the lowest sheep losses of the six farmers in the first outbreak and perceived the severity of the impacts to be at the lowest level, but felt uncertain about the risks of repeat infections and future disease outbreaks.

**Conclusion:**

Severity of farmer perceived impacts of SBV infection was generally high; farmers’ detailed descriptions of their experiences during the outbreak, and perceptions of the impacts on ewe and lamb welfare, financial performance and their emotional well-being, captured during semi-structured interviews, are reported for the first time. Variation in severity of impacts appeared not to be directly related to the number and proportion of lambs stillborn or that died in the first week of life and the overall reduction in percentage of lambs reared for sale. Qualitative interview data taken together with quantitative data on recorded flock performance suggested multiple factors and variable effects of SBV in flocks were likely to have contributed to, and variably influenced, the severity of impacts perceived by farmers. Uncertainty about the safety, efficacy and use of the vaccine so close to the next early breeding season when it was first authorised in May 2013, and the risks of repeat SBV infections and future disease outbreaks for farmers who decided not to vaccinate their flocks may have added to the impact on farmers’ emotional well-being. Reductions in lamb rearing percentage appeared to be higher in flocks that artificially inseminated ewes in synchronised oestrus in July 2012 than in those that mated ewes naturally in spontaneous oestrus in June 2012. These findings are important and suggest that recorded lamb losses and reduction in rearing percentage should not be used as proxy measures of the severity of impacts of SBV infection on farmers and sheep flocks. Further outbreaks have occurred in the UK in 2016/2017 and 2021/2022 and it is expected this pattern of virus circulation and disease re-emergence will be repeated every 3–6 years. Flocks remain at-risk of future SBV infection and, in high risk flocks, of severe impacts on animal health and welfare, flock financial performance and farmers’ emotional well-being. Further research is needed to explore farmers’ future risk perceptions, uncertainty and decision-making around preventive vaccination, and to explore the potential for more rational vaccination programmes based on active arbovirus (SBV and Bluetongue virus [BTV]) surveillance systems.

**Application:**

These findings will be of interest to all stakeholders in the sheep industry e.g. farmers, veterinarians, advisers, researchers, welfare organisations, pharmaceutical companies, the UK Government, industry levy boards and other research funding bodies. The study offers new insights into the impacts of SBV infection in sheep flocks, particularly in production systems dependent upon early breeding (so called ‘out of season breeding’) overlapping with periods of peak midge activity and circulation of SBV in which risks of high impacts appear to be greater. Other studies are needed to investigate further possible associations between variability in reproductive outcomes and factors such as breed (not reported here) and timing and method of breeding (natural mating or artificial insemination; at a spontaneous or synchronised oestrus). Research is needed to better understand farmers’ decision-making around SBV vaccination and to investigate the potential for more rational vaccination programmes based on early warning systems, such as national or Europe-wide arbovirus surveillance systems.

## Introduction

In October 2011, Schmallenberg virus (SBV), a novel Orthobunyavirus, was identified as the cause of a mild acute infection in cattle that had emerged in summer and autumn 2011 in the border region between Germany (North Rhine-Westphalia), the Netherlands and Belgium; clinical signs included reduced milk production, fever and diarrhoea (Hoffmann et al., 2012). In November 2012, SBV infection of pregnant ruminants was associated with abortions, stillbirths and the birth of offspring with congenital malformations (Gibbens, 2012; Herder, Wohlsein, Peters, Hansmann, & Baumgärtner, 2012; Lievaart-Peterson, Luttikholt, Van den Brom, & Vellema, 2012). Investigations identified that acute SBV infection in naïve ewes can go unnoticed, but may result in early embryonic death, repeat mating and increased proportion of barren ewes; whilst infection of the foetus at a critical stage of pregnancy (days 30–50 in sheep (Parsonson, Della-Porta, & Snowdon, 1977)) can cause abortions, stillbirths and a range of congenital malformations in newborn lambs known as the arthrogryposis and hydrancephaly syndrome (AHS) (Afonso et al., 2014; Anonymous, 2012a; Lievaart-Peterson et al., 2012).

The spread of SBV throughout Europe has been well documented (Afonso et al., 2014; EFSA, 2014). The first reports of SBV infection in lambs with congenital malformations were reported in the Netherlands in late November 2011 (van den Brom et al., 2012 cited by Lievaart-Peterson et al., 2012), in Belgium in December 2011 (Martinelle, Dal Pozzo, Gauthier, Kirschvink, & Saegerman, 2014), and in France (Dominguez et al., 2012) and the UK in January 2012 (Anonymous, 2012b). However, with surveillance studies focused on identifying the spread of SBV, there was a lack of data on within-flock impacts of the first outbreaks of infection (Afonso et al., 2014; EFSA, 2014). Despite a lack of data, in summer 2012 the UK Government described Schmallenberg as a ‘low impact disease’ (Simmons, 2012a). This assessment did not fit well with our veterinary experience of outbreaks in early lambing flocks in South West England in November and December 2012 (M. Glover & Blake, 2012). Subsequently, the UK Government acknowledged that within-flock impacts had been variable and were likely to be higher in early lambing flocks (Simmons, 2012b). This assessment was later supported by national impact studies carried out in the UK (Harris et al., 2014; Stokes et al., 2018), and elsewhere in Northern Europe (Dominguez et al., 2012; Luttikholt et al., 2014; Saegerman, Martinelle, Dal Pozzo, & Kirschvink, 2014).

This study aimed to add to research on within-flock impacts of SBV infection by: firstly, quantitatively describing the impacts perceived by farmers of the first outbreaks of SBV on reproductive performance and production outcomes in early lambing flocks in South West England; secondly, qualitatively describing perceptions and experiences of the physical signs of SBV that farmers observed at lambing time, and impacts on flock welfare, production and financial performance, and on farmers’ emotional well-being (reported previously by C. J. Phythian and Glover (2019)); thirdly, quantitatively evaluating the farmer perceived severity of those impacts using a simple scoring system; and finally, describing farmers’ perceptions of the risk of future outbreaks of SBV disease and preventive strategies including SBV vaccination.

## Methods & Materials

### Study design

Farmers of seven small pedigree and purebred early lambing sheep flocks in South West England (counties of Devon, Cornwall and Somerset), with clinically confirmed cases of SBV infection in AHS affected lambs, who had previously participated in a serological SBV survey (Mike Glover & Blake, 2013), and who we expected had maintained detailed lambing records, were invited to participate in the current study through contact with Torch Farm and Equine Ltd, a veterinary practice based in North Devon. Serological sampling in May and early June 2013 had identified that a substantial proportion of ewes (86.7%, range: 34.9–93.6%) in these flocks had been exposed to SBV infection in the second wave of virus circulation in the UK in summer 2012 and had seroconverted (Mike Glover & Blake, 2013). They were considered to be amongst the first flocks in South West England to be affected by SBV infection. The small size of the flocks, nature of early lambing to achieve high prices for slaughter lambs and breeding stock, and loss of valuable pedigree breeding animals were of particular interest in understanding the perceived and recorded impacts of the first outbreaks of infection on these farms.

A mixed-methods approach was used to gain new insights into farmers’ observations and perceived impacts of SBV infection. Each farmer was invited to participate in a semi-structured face-to-face interview conducted at a time and a location of their choice. Participation was voluntary; one of the seven farmers declined the invitation to be interviewed, not wishing to relive the experience. Prior to undertaking the study, interview questions were pilot tested on one commercial sheep farmer to ensure interviews would take approximately 30 minutes. Interviews were conducted during August to October 2013. Before each interview, informed, written consent of participants was obtained. The study was approved by the University of Bristol, Faculty of Medical and Veterinary Science Research Ethics Committee (number 3283).

### Data collection

Data were collected on participant demographics: farm type, and flock size, purpose and breed. Farmers were asked the same series of open questions (see Supplementary material S1 – Interview Questioning Guide) to capture new insights (qualitative data) on their experiences of SBV infection, including what they had witnessed in their flocks during the early lambing period between November 2012 and January 2013; and what changes they had made following their first experiences of SBV infection including; had they changed the timing of their breeding season, and had they vaccinated all or part of their early lambing flocks, or considered vaccination, to prevent future outbreaks of SBV. All six interviews took place around the farmhouse kitchen table. Interviews were conducted by a veterinary researcher (CJP) who was unfamiliar to the participants and had designed the interviews to capture useful data, and ensured the conversation avoided diverging into a veterinary clinical or advisory discussion (Gill, Stewart, Treasure, & Chadwick, 2008). A familiar veterinarian (MJG) was present to put respondents at ease, build confidence and rapport around discussing potentially sensitive topics with an unfamiliar visitor (CJP).

At the end of the interview, each farmer was asked to score (quantitative data) the severity of perceived negative impacts on flock welfare, financial performance and their emotional well-being on a scale of 1 to 5 (1 = no impact, 5 = high impact) using the approach of Harris et al. (2014). Quantitative data on flock reproductive outcomes, based on farmer-maintained records, were collected at the end of the visit and during follow-up contact with the farmers, in order to evaluate flock reproductive performance and key production outcomes. Flock data on the 2012/2013 early lambing production year, and where available for up to 3 years prior, were collected to ascertain; breeding and lambing start and end dates, ewe to ram ratio, number of ewes mated, number of barren ewes, number of lambs born alive and stillborn, number of ewes and lambs clinically affected by SBV, number of assisted lambings including embryotomies and caesarean sections, recorded ewe and lamb mortality rates, and rearing percentage (number of lambs reared relative to the numbers of ewes mated in the early lambing flock in 2012 expressed as a percentage).

### Data analysis

Qualitative (interview) data were captured as individual digital voice recordings. Recordings were transcribed verbatim. Transcription responses were double-checked against the voice recording for consistency before responses were examined using a Thematic Analysis approach (Braun & Clarke, 2006). Farmer responses were examined and manually coded by one researcher (CJP) to identify broad themes. Coding was later checked by a second researcher (MJG). Where any discrepancy was identified, the descriptions were reviewed and a consensus reached.

Farm records were descriptively analysed by a second researcher (MJG) to calculate key reproductive and production outcomes, including scanning percentage, rearing percentage, ewe and lamb overall mortality and mortality attributed to SBV infection. The approach of Barrett, O'Neill, Sammin, Clegg, and More (2015) was applied to calculate a rearing factor, the ratio of rearing percentage for 2012/2013 to the baseline rearing percentage for each flock. The baseline used for comparison was either a flock-specific average based on retrospective farm data prior to the 2012 / 2013 production year; or, if retrospective data were lacking, on an industry benchmarking figure for early lambing flocks (EBLEX, 2009).

## Results

All six participants were of white British ethnicity, three male and three female, aged between 30–60 years. The numbers of ewes mated on these farms in the 2012 / 2013 early breeding season varied from 27–320 as previously described (C. J. Phythian & Glover, 2019). Study farms reared pedigree or purebred sheep breeds including Charollais and Polled Dorset. To maintain anonymity, flock location and breed are not presented.

### Recorded impacts on reproductive performance (quantitative element)

Key flock performance indicators and impacts of SBV infection on the 2012 / 2013 early lambing production year are presented in Tables 1 and 2. Compared to previous years, recorded barren ewe rates were higher in three flocks (A, B and F) and lower in one (E), lamb losses from pregnancy scanning to sale were higher in three flocks (B, E and F), and lamb rearing percentages were lower in five flocks (A, B, and D–F). Two farmers (A and E) attributed higher ewe mortality rates around lambing to SBV. Five farmers (A–D and E) reported that higher levels of lambing assistance were required to deliver SBV affected lambs; these observations were supported by farm records for four flocks (A, B, D and E). Of all lambs born, an overall average of 21.2% (207/975; range: 13.7–42.6%) were either stillborn or died within a week of birth and 15.0% (146/975; range: 4.1–42.6%) were born dead or died due to SBV infection.

Rearing percentage was calculated for the 2012/2013 production year for five (A, B, and D–F) of the six flocks in which the relevant data had been recorded by farmers (see Table 1). In comparison with baseline figures for earlier years, or an industry benchmark (EBLEX, 2009), rearing percentages were reduced in flocks B and D (by a factor of 0.91 and 0.88 respectively) and severely reduced in flocks A, E, and F (by a factor of 0.63). Details of the breeding management used and reductions in rearing percentage are presented in Table 1: rearing percentages in flocks B and D, where ewes were naturally mated to lamb in November, were reduced by ~10% compared to baseline figures; whereas, in flocks A, E, and F, where ewes were inseminated at a synchronised oestrus to lamb in December, rearing percentages were reduced by 37%. Lack of recorded data for flock C prevented calculation of a rearing percentage.

### Clinical signs of SBV infection (qualitative element)

The main themes regarding the clinical signs of SBV infection reported by farmers in ewes and lambs were (i) lack of premonitory signs in pregnant ewes until around lambing, (ii) higher than normal levels of dystocia, protracted births and need for lambing assistance to deliver congenitally malformed lambs, and (iii) the high prevalence and severity of congenital deformities in lambs. Individual quotes were selected to illustrate these broad themes, as presented below.

### Lack of premonitory signs

All participants reported an absence of premonitory signs of SBV infection in ewes from early to late stages of pregnancy:

‘I wouldn't have said anything was wrong with them. They were scanned normal. Once we'd gone through scanning we didn’t even think of Schmallenberg again.’ (Farmer E).

Signs of infection were only apparent at the time of lambing as farmer C recalled:

‘I was the only one lambing the sheep. It was completely different. No signs whatsoever, you'd have a good one then a bad one. You can tell soon as you put your hand in and you get that back, you know something is not right’.

Retrospective analysis of farm records indicated that two flocks (C and F) had experienced repeat breeding during the 2012 early breeding season and three flocks (A, B, and F) had a considerably higher proportion of barren ewes (Table 1) than in previous lambing seasons. A large proportion of ewes mated (n = 128, 47%) were barren at lambing in flock C, but no baseline figure could be calculated for comparison (Table 1). In hindsight, two farmers (B and D) reported that ewes appeared to have been particularly affected by biting midges in the late summer of 2012, but recalled no apparent premonitory physical signs of SBV infection in ewes.

### Dystocia and protracted lambings

All but one farmer (F) recalled difficult or protracted lambings and the need for an unusually high degree of lambing assistance:

‘I think we lambed nearly everything. Probably three of four of them we didn't lamb. Most of them had something going on. Normally we lamb, half a dozen at most. We wouldn't normally lamb many at all.’ (Farmer E).

Farmers elected to give earlier assistance at lambing with increasing awareness of the issues:

‘I did pull out more than I normally did. Suppose, perhaps I started going in more than I would normally, once I knew what was going on.’ (Farmer C).

Rarely were malformed lambs born without assistance:

‘You had to try to get, well all of them out. There was some that you'd come down and there was a couple of deformed lambs born, which lambed themselves. Ninety percent of affected ewes had to be lambed.’ (Farmer D).

A consistent feature reported by farmers was the observation that the birthing process took longer than normal:

‘They would quite take [*sic.*] a long time to lamb, they would probably take up to 24 hours to lamb, to get something presented correctly.’ (Farmer E).

The extent of dystocia, and their first experience of lambing ewes giving birth to malformed lambs, took farmers by surprise and gave cause for some self-doubt:

‘I mean the first ewe I lambed, I didn't realise what was happening and I thought what's wrong with me, I can't get this leg forward, I cannot and […] I'm usually not that bad I can get that leg forward but I thought what's wrong with me, and when it came out I knew it looked like something was wrong[...] that's my experience of it.’ (Farmer D).

Almost all farmers dealt with dystocia cases by themselves. Three farmers (B, C, and E) reported undertaking embryotomies and one (E) initially required veterinary assistance to manage cases of dystocia and deliver malformed lambs, including carrying out a caesarean section. Farmer E later became reluctant to seek veterinary support as farm staff became familiar with the abnormal presentations of malformed lambs, and after experiencing poor outcomes even when dystocia cases received veterinary assistance:

‘We had the vet[...] probably say six[…] or seven times, can't remember now[…] We had one caesarean. Had one we cut up, which was a disaster, ‘cause everything died then. We only lost one ewe[…] that was during one of these embryotomies[…] we'd leave them a fair while, once we knew what we were dealing with because one we didn't want vets visits, and we were trying to just see if we can deal with it ourselves.’ (Farmer E).

### Congenital deformities

Farmers described a range of severe congenital physical, behavioural and neurological abnormalities in newborn lambs; particularly striking features were spinal deviations (torticollis, kyphosis and scoliosis).

‘You'd occasionally put your hand around a jaw to pull it around and it, yeah, it was not pleasant. Some of the worst ones had very, very roached backs[…] really arched so you were struggling to move them around. The necks were the things that were quite prominent. I would say about half of them had funny necks, and nearly all of them had odd limbs. I think virtually all of them had twisted or deformed legs in some way.’ (Farmer E).

**Figure 1:**
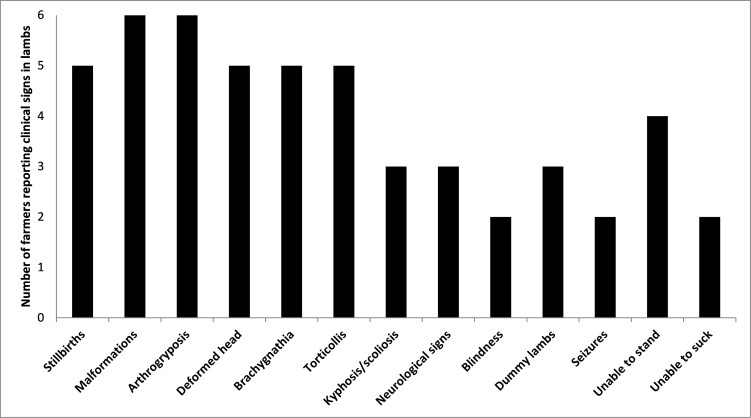
Number of flocks (n = 6) reporting specific clinical signs associated with congenital SBV infection in lambs born during the early lambing season from November 2012 to January 2013

Commonly reported signs in SBV affected lambs, observed by at least five of the six farmers, were: arthrogryposis, torticollis, brachygnathia, deformed skulls and stillbirths (Figure 1); less commonly reported, by three or fewer farmers, were kyphosis/scoliosis, and neurological signs including blindness, seizures and ‘dummy’ lambs; see Figure 2a and 2b, and video clips of neurological signs shown by two liveborn SBV-affected lambs in flock D provided as supplementary material (Clip 1 – Liveborn Schmallenberg affected lamb with seizures; Clip 2 – Liveborn Schmallenberg affected blind ‘dummy’ lamb).

**Figure 2a:**
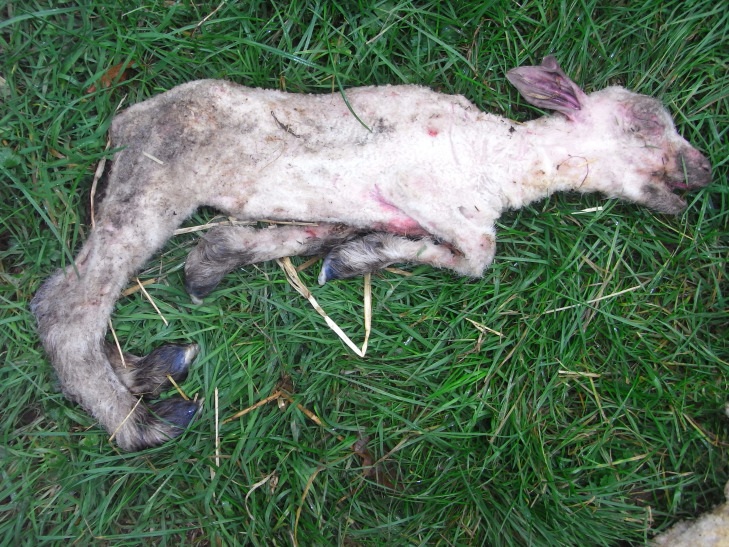
Lamb with congenital malformations: arthrogryposis, kyphosis, skeletal muscle hypoplasia and brachygnathia

**Figure 2b:**
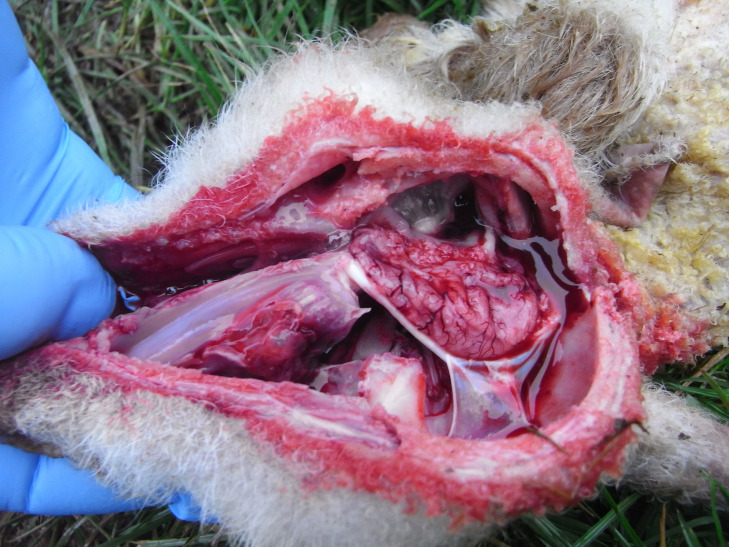
Hypoplasia of the central nervous system: hydrancephaly

A wide variety of malformations and abnormalities were observed:

‘[We] had a whole range, screwed up, mummified ones, then big lambs lying there and didn't stand up. Some had their necks bent around, some were just first, funny jaws and, they were like little aliens really. Some were really big, some really small. They were all different. I had one that really sticks in [my] mind, I don't know if he was blind, but he went around the pen shouting and wouldn't suck.’ (Farmer C).

Many of the affected lambs were born alive but with apparent reduced cognitive ability:

‘We had one or two that were erm, their brain didn't seem to be three-quarters there and we used to have to work quite hard to let them suck. We had half a dozen like that, not many. It was like we had some totally deformed ones, laying right back and around, totally still. A small percent were nearly there but not quite[...] there were a few born dead but I would say about three-quarters of them were born alive.’ (Farmer D).

However, not all lambs from the same litter were physically affected, which surprised even some of the most experienced shepherds:

‘Very often the first lamb would be born fine, and then there'd be a longer than normal time before they tried to get settled and you know then that there's something happening don't you? Then you'd erm, obviously examine the ewe and you'd find that the second lamb more often than not would be there, with legs all wrong, leg back. My Dad couldn't believe when he came, he couldn't believe one could be born alright and the other could be born so wrong.’ (Farmer D).

Farmers were not unfamiliar with the occasional birth of lambs with congenital malformations in previous years, but what struck them most was the intensity and regularity with which malformed lambs were born:

‘We weren't losing lamb after lamb[…] but we had deformed lambs every single day.’ (Farmer B).

### Perceived impact on flock welfare (qualitative element)

The main themes identified regarding farmers’ concerns and perceptions for flock welfare, were; (i) the severe impact of dystocia and physical trauma, damage and death of ewes associated with the birth of malformed lambs and their own interventions in these cases, and (ii) the impact of congenital malformations and neurological deficits on the viability of lambs at birth and ability to survive the neonatal period.

### Impact of dystocia on the welfare of ewes

Ewes in five of the six flocks (not on farm A) required varying levels of farmer and veterinary assistance to deliver malformed lambs leading to concerns about both short-term effects on ewe health and welfare, and long-term effects on future productivity:

‘It was traumatic, probably, ‘cause the lambings were so difficult. It will be interesting what the scanning comes out [with] this time, if it's affected them in the future. A lot of them were battered and bruised so it has got to have affected them the welfare side of it [*sic.*].’ (Farmer E).

‘I did pull out more than I normally did. Suppose, perhaps I started going in more than I would normally, once I knew what was going on. I got them all surprisingly easy, once I knew. Some of the big ones they just fell apart.’ (Farmer C).

Farmers were in a dilemma as to how best to deliver malformed lambs without causing unnecessary pain or suffering to ewes.

‘I just wanted to get lambs away without too much stress to the ewe, which was not easy. You can't draw the legs out [if] they are all twisted back.’ (Farmer A).

The higher level of dystocia resulted in an unusually high ewe mortality rate in two flocks (A and E):

‘You wouldn't lose that many sheep during the normal lambing process would you, not before they've lambed.’ (Farmer A).

### Impact of congenital malformations and neurological deficits on the welfare of lambs

Farmers evaluated the welfare of affected liveborn lambs weighing-up their ability to survive. Severely affected liveborn lambs were euthanised on farm or at local hunt kennels. Other farms had to deal with a higher than usual number of stillborn lambs:

‘[…] we had to put down some ‘cause they couldn't suck or survive or even get up. There would be more, the majority, that[…] had to be put down. Nothing was ever similar. They were all completely different. [The…] only thing that compared to that [SBV] was abortion, but not in the later ones [the later lambing flock].’ (Farmer C).

Affected liveborn lambs needed more care than expected:

‘We had one or two that were erm, their brain didn't seem to be three-quarters there and we used to have to work quite hard to let them suck. We had half a dozen like that, not many, but we tried to rear them all. A small percent were nearly there but not quite. Most of the lambs were born alive, there were a few born dead but I would say about three-quarters of them were born alive.’ (Farmer D).

Other farmers had less success and expressed disappointment that lambs died despite their best efforts to avoid euthanasia:

‘A couple [of lambs] never stood up, there was one single that we kept in the shed and he was fine as long as nothing disturbed him, and I let the ewe out into the main part of the shed and the lamb was totally lost and he, he didn't suck again and he just died.’ (Farmer B).

### Perceived financial impact (qualitative element)

Three main themes were identified regarding the perceived financial losses: (i) reduction in lamb sales compared to previous years; (ii) loss of genetic material; and (iii) higher rearing costs than in previous years.

Farmers in the study perceived the reduction in lamb sales to be approximately £100/lamb lost based on the value of lambs destined for slaughter:

‘It was horrific. For the sheep it would be as somewhere, if I was selling those fat, those 20 [lambs] that died, it would be £2000[…]’ (Farmer E).

‘We lost 75 lambs – that's what we make isn't it, £7500.’ (Farmer D).

However, these pedigree and purebred sheep farmers also felt they had lost valuable genetic material, built up over many years, in breeding stock and potential ewe replacements that died due to SBV:

‘[...] but some of those would be breeding stock[…], so somewhere near £5000 [*sic.*] [in lost profit] and some of those would be ewe replacements, difficult to put a figure on those – part of a generation that we are going to miss.’ (Farmer E).

‘The three ewes that I lost[…] It's not so much of the financial is it? It's the breeding[...]’ (Farmer A).

Veterinary and medicine costs outweighed potential profits in 2012/2013:

‘Probably spent £1500 on vet visits and drugs because we were giving a lot of drugs to make them live.’ (Farmer E).

Whereas, feed costs were of more concern for others:

‘My costs of living lambs isn’t going to cover the costs for keeping lambs. Not going to cover the feed bill. Fat lambs[…] 70% less than normal. It's quite frightening really.’ (Farmer C).

Workload and resources needed during lambing were increased but profits were reduced:

‘Since we lost 24 [ewes] that year and not all because of the disease you still have to do the same amount of work to rear fewer lambs and employ the same staff.’ (Farmer B).

### Perceived impacts on farmers’ emotional well-being (qualitative element)

Results of thematic analysis of the qualitative interview data on perceived impacts on farmers’ emotional well-being have been reported in detail previously (C. J. Phythian & Glover, 2019). Briefly, three themes emerged from the interviews: (i) emotional highs and lows are part of a normal lambing season, e.g. ‘when things are going great, there’s no better thing to be doing than lambing really’ (Farmer B), and ‘To me that’s breeding, that’s what we do, they live or they don’t live[…]’ (Farmer C); (ii) negative emotions and memories associated with the first outbreaks of this novel disease, e.g. ‘I was just upset, just upset about the losses isn’t it[…] any losses are, you know[...] especially when it’s the ewes. I think lambs[...] you lose lambs don’t you, but when it comes to the ewes you kind of feel a bit more touched.’ (Farmer A); ‘I think I just remember feeling a little bit more stressed than normal. That’s how it works when you’re under a little bit of pressure, I mean normally you cope with things better at that time.’ (Farmer D); and ‘It affects your mental health. It was difficult, you didn’t want to, you just knew you had to go outside again. It was very depressing at the end, cause you know every time, virtually, you would have a problem.’ (Farmer E); and (iii) resilience and coping with these unexpected disease outbreaks, e.g. ‘I was just thinking it’s not in my children, thank goodness it’s not in my children.’ (Farmer C), and ‘speaking to friends and neighbours they had something much worse than we had.’ (Farmer B).

### Severity of farmer-perceived impacts on flock welfare, financial performance, and farmers’ emotional well-being (quantitative element)

**Figure 3:**
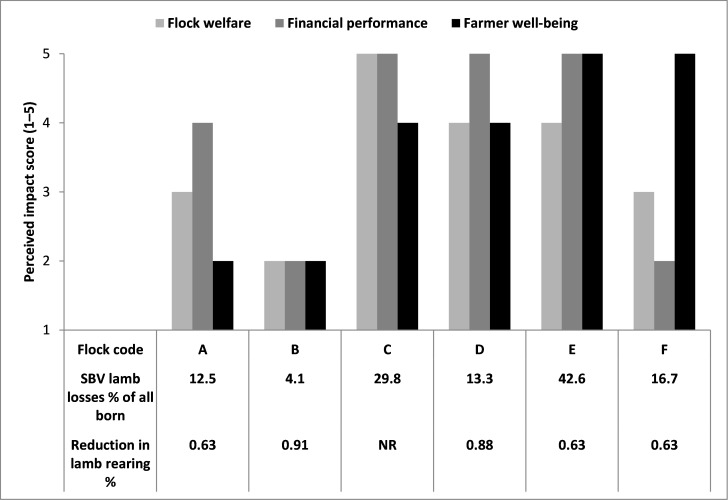
Farmers’ self-appraised severity scores of the negative impact of Schmallenberg virus on flock welfare, financial performance, and their emotional well-being (rated from 1 = no impact; to 5 = high impact (Harris et al., 2014) by SBV lamb losses and rearing percentage reduction factor (NR = not recorded) in six pedigree or purebred flocks (labelled A to F) during the 2012/2013 early lambing season

Farmer-perceived impacts are presented as self-appraised categorical severity scores in Figure 3. All farmers perceived negative impacts on flock welfare, financial performance, and their own well-being. Overall severity was generally high, although self-appraised scores varied widely between farmers: for the impact of SBV on animal welfare the median severity score was 3.5/5 (range: 2–5); on financial performance the median score was 3.5/5 (range: 2–5); and on farmers emotional well-being the median score was 4/5 (range: 2–5). Farmers C and E, with the highest overall self-appraised severity scores, recorded the highest neonatal lamb losses (29.8% and 42.6% of all lambs born, respectively) due to SBV; whereas farmer B, with the lowest overall severity scores (level 2), recorded the lowest neonatal lamb losses (4.1% of all lambs born).

Farmers C, D, and E perceived the impact of SBV infection on flock welfare to be at the two highest levels (scores ≥4); flocks D and E (not recorded for flock C) had recorded a high proportion of ewes delivering SBV affected lambs and requiring assistance to give birth, and all three flocks (D, E and C) had recorded high proportions of lambs stillborn or dead due to SBV during the first week of life.

Severity of farmer perceived impacts on financial performance varied widely, even between farmers experiencing similar lamb losses due to SBV. For example, farmer F, who recorded losing 16.7% (7/42) of all lambs born due to SBV during the first week of life and losing 35.3% (18/51) of lambs between scanning and sale (Tables 1 and 2), perceived the severity of the impact on financial performance to be at the lowest level (score 2) despite an overall reduction in percentage of lambs reared by a factor of 0.63 in 2012–13 (Table 1); whereas, farmer A, with a similar level 12.5% (5/40) of recorded newborn lamb losses due to SBV , and the same reduction in percentage of lambs reared, perceived the financial impact to be high (score 4). In addition to similar lamb losses and reductions in lamb rearing percentage as flock F, flock A also lost three ewes at lambing due to SBV, 9.1% (3/33) of those mated, which flock F did not.

There were two relative extremes in self-appraised severity scores for farmer-perceived impacts on emotional well-being ranging from the lowest level (score 2) of farmers A and B, to the two highest levels (score ≥4) of the four farmers, C–F. Farmer B had experienced the lowest lamb losses of the six farmers and a low proportion of ewe losses due to SBV, no ewes required assistance to deliver malformed lambs, and perceived the negative impacts on animal welfare and flock financial performance to be at the lowest level (score 2); whereas farmers C–F had experienced higher lamb losses, and variable other effects of SBV e.g. on ewe mortality, dystocia and proportion of ewes requiring assistance to deliver malformed lambs (up to 92% of ewes [23/25] in flock E).

### Perceived future risks of SBV infection and changes made to prevent future disease (qualitative element)

Farmers had weighed up the risk of repeat infection with SBV during subsequent pregnancies either independently or in consultation with their local veterinarian; and with the benefit of information on levels of flock immunity based on blood test results and flock SBV seroprevalence (Mike Glover & Blake, 2013) following the first outbreaks of disease in these flocks, considered possible changes to the management of their flocks and deployment of SBV vaccine to help reduce risks of future outbreaks. Three themes emerged during the interviews: (i) decision to continue sheep farming, (ii) commitment to the current breeding system and considerations around delaying the timing of the breeding season to avoid the critical period of pregnancy for congenital malformations coinciding with the peak of midge vector season in summer and early autumn, and (iii) use of flock SBV vaccination to prevent future disease outbreaks.

### Decision to continue sheep farming

All six farmers had decided to continue sheep farming despite their experiences during the first outbreaks of SBV infection, although one farmer suggested this decision would be reconsidered if repeat outbreaks of disease occurred in future:

‘It affected me a little bit, but not, not so bad, if it [SBV] was going to happen every year, I wouldn't do it [breed sheep] again.’ (Farmer C).

### Committed to the breeding system

All six farmers were committed to breeding and lambing their pedigree and purebred flocks early to produce lambs in November and December to allow lambs time to be sufficiently well grown for the breeding stock sales (of ram and ewe lambs) in the summer and early autumn following birth, or for sale as ‘finished’ slaughter lambs when they would be most likely to achieve high prices:

‘Kind of committed to the system now, you can't change the whole system just for one blip.’ (Farmer C).

‘We haven't made any changes at all. We've carried on as normal. When I want to sell ram lambs, I want to sell around 8 to 9 months old so I have to lamb that time [early December].’ (Farmer E).

‘No [we haven’t made any changes since that experience of SBV], we still lamb at the same time [November] and we didn’t vaccinate last year [prior to 2013 early breeding season].’ (Farmer B).

### Changing the timing of the breeding season

Farmers were aware that the first outbreaks of disease due to SBV in the UK had occurred in early lambing flocks; later (spring) lambing flocks had been largely unaffected, indeed five of the six farmers (not E) not only had an SBV affected early lambing flock but also had an unaffected spring lambing flock. It was understood that in the absence of a safe and effective SBV vaccine an option to reduce the risks of future disease outbreaks was to delay the start of the breeding season so as to avoid the peak midge-vector season and exposure of ewes to SBV infection coinciding with the critical stage of pregnancy in ewes (Anonymous, 2013). One farmer (A), decided to delay the start of the subsequent breeding season for the early lambing flock by 1 month, rather than vaccinate:

‘No, I didn’t alter my decision [about vaccination] when I saw the blood results [around a quarter of ewes in the early lambing flock, and half of the yearling replacement ewes, were seropositive for SBV…] I had already decided to lamb 1 month later.’

### Use of flock SBV vaccination to prevent future disease outbreaks

There had been an awareness of the rapid development, authorisation and introduction to the market of a SBV vaccine in May 2013, a short time before the subsequent early breeding season, offering farmers an immunological option to control SBV infection and prevent disease:

‘You have got the opportunity to vaccinate, and that is your choice, it’s not as if we are without vaccine, which is a plus because it was brought in on pretty fast speed [*sic.*]... you have the choice.’ (Farmer B).

Three of the six farmers (C, D and F) had decided to vaccinate ewes and replacement females in their early lambing flocks:

‘We vaccinated the following year [prior to the 2013 early breeding season], that was the major thing [we changed after the experience of SBV].’ (Farmer D).

Farmer C had vaccinated the early lambing flock but decided not to vaccinate the spring lambing flock, appearing to highlight the possibly one-off nature of the first outbreak and perceived lower risk of severe impacts in later lambing flocks:

‘I vaccinated the first ones [early lambing flock] I suppose, but I haven't bothered with the later flock.’

Farmers who decided to vaccinate, vaccinated their breeding ewes but not their rams, most likely following advice from their veterinarian and the manufacturer that the safety and efficacy of the SBV vaccine had not been established in breeding males; one farmer had not considered vaccinating rams:

‘We just vaccinated the ewes, we didn't even think about doing the rams.’ (Farmer F).

Three farmers (A, B, and E) decided not to vaccinate their flocks. Farmer E had concerns about using the new vaccine so close to the start of the subsequent breeding season and the possibility of adverse effects on the outcome of an artificial insemination (AI) programme. This farmer had been somewhat reassured by veterinary advice, and the results of serological testing following the first outbreak indicating a high level of flock immunity, that there was a low risk of repeat infection during the subsequent pregnancy (mean flock SBV seroprevalence had been ~87%) (Mike Glover & Blake, 2013):

‘We discussed it with the vet, it [SBV serological testing] indicated that we'd had it quite severely. He [the vet] said if the vaccine doesn't come in time the risks, hopefully, are minimal. When the vaccine came out it was quite close to our AI date. When they were saying 3 weeks, some saying do it up to 4 weeks before [breeding], I said I'm not even going to take the risk [of vaccinating],’ (Farmer E).

Farmer A delayed the start of the subsequent breeding season by 1 month to reduce the risk of a further disease outbreak. Farmer B had decided neither to vaccinate, nor to delay the start of the breeding season, but expressed some uncertainty about future risks of repeat SBV infections and disease outbreaks:

‘No, we still lamb at the same time and we didn’t vaccinate last year[…] It's [risk of future SBV infection] just a concern looking forward. Again you have got the opportunity to vaccinate, and that is your choice.’

## Discussion

Two previous studies have assessed the impacts of SBV infection on British sheep farms following outbreaks of disease during the 2011/2012 (Harris et al., 2014) and 2016/2017 (Stokes et al., 2018) lambing seasons. However, to our knowledge, this is the first time that farmers’ detailed qualitative descriptions of SBV affected lambs and ewes; in-depth quantitative evaluations of key production indices for flocks experiencing outbreaks of disease during the 2012/2013 early lambing season in the UK; qualitative and quantitative analysis of farmers’ perceptions of the impacts of SBV on animal welfare, flock financial performance and their emotional well-being; and perceptions of the risk of future outbreaks of SBV disease and preventive strategies including SBV vaccination, have been presented in a mixed-methods study.

The clinical signs most commonly observed in SBV affected lambs were; stillbirths, arthrogryposis, deformed heads and stiff necks (torticollis); other spinal deviations (kyphosis / scoliosis) and neurological signs were observed less commonly, as reported by others (Dominguez et al., 2012; Harris et al., 2014). Farmers’ descriptions were consistent with a clinically-silent pattern of disease in ewes in the acute phase of SBV infection in sheep in summer 2012, as described elsewhere (Peperkamp et al., 2015; Saegerman et al., 2014). However, with the benefit of key flock reproductive performance indicators for the 2012/2013 early lambing season and similar data for previous seasons for comparison, there appears to have been evidence to suggest that there were effects of acute SBV infection in summer 2012 on reproductive events other than during the critical period of pregnancy for congenital malformations in lambs; e.g. scanning percentage was lower in three of the four flocks where data were available and similar in the other, percentage of ewes barren at scanning was higher in the same three flocks and similar in the other, and in five of the six flocks ewes that were scanned in-lamb lost lambs (aborted) after scanning and were barren at lambing.

In this study, an overall average of 21.2% (207/975, range: 13.7–42.6%) of lambs born were either stillborn or died within a week of birth and 15.0% (146/975, range: 4.1–42.6%) were born dead or died due to SBV during the 2012/2013 early lambing season; substantially higher losses than an accepted industry benchmark of 5.2% for average lamb mortality in early lambing flocks (EBLEX, 2009), and higher than findings from other impact studies (Harris et al., 2014; Stokes et al., 2018; Domingues et al., 2012; Saegerman et al., 2014; and Luttikholt et al., 2014).

The first survey of the impacts of SBV on British sheep farms following the first wave of virus circulation in southern England in 2011 and outbreaks of disease during the 2011/2012 production year found an average of 10.4% of all lambs born died within 1 week of birth on SBV confirmed sheep farms and 5.5% of lambs were born *malformed* (Harris et al., 2014). The second survey of British sheep farms, following recirculation of virus and recrudescence of disease during the 2016/2017 production year, similarly found 9.1% of all lambs born died within a week of birth on SBV confirmed farms (Stokes et al., 2018). Elsewhere, in France, almost 13% of lambs in SBV infected flocks were born dead or died within 12 hours of birth during the first outbreak, and 10% showed congenital malformations typical of SBV (Dominguez et al., 2012); similarly, in Belgium, 13.2% of lambs were stillborn or died at birth and 10.1% had deformities in SBV affected flocks (Saegerman et al., 2014); and in the Netherlands, in a case-control study, 13.9% of lambs were born dead or died prior to weaning in case flocks and 5.2% were born with congenital malformations (Luttikholt et al., 2014).

Differences in findings between this and other impact studies are likely to reflect differences in the samples (sheep flocks or farms) surveyed, sample size and other methodological differences, and epidemiological differences between waves of SBV infection in the UK in 2011/2012, 2012/2013 and 2016/2017. The current study was small, farmers with pedigree and purebred early lambing flocks were purposively selected, recorded and perceived impacts were evaluated at flock level and compared reproductive performance in the affected year with previous years, and all flocks lambed early over a relatively short period between 5 November 2012 and 31 January 2013. The two previous British impact studies (Harris et al., 2014; Stokes et al., 2018) were larger, SBV confirmed sheep farms were purposively selected for inclusion whilst others responded to an online survey, impacts were estimated at farm rather than flock level and compared reproductive performance indicators between groups of farms on which SBV had been (laboratory) confirmed, was suspected or not suspected by farmers, and the sheep farms included lambed over extended periods from September 2011 to June 2012, and from October 2016 to June 2017 respectively. As a result, findings of the two previous studies and this study may not be directly comparable. Neither of the previous studies was able to collect data from previous production years for comparison with performance in the SBV affected year, and neither was able to assess overall impacts on productivity and financial performance of affected flocks. Acute SBV infection, whilst not clinically apparent in sheep, can have severe negative consequences on early reproductive events resulting in poor conception rates, early embryonic deaths, repeat mating, high barren rates and early abortions (Dominguez et al., 2012; Lievaart-Peterson et al., 2012; Luttikholt et al., 2014; Saegerman et al., 2014); however, no evidence of early gestational effects were found in the two previous British studies that compared barren rate and lambing percentage between SBV confirmed, suspected and not suspected sheep farms (Harris et al., 2014; Stokes et al., 2018). Stokes et al. (2018) suggested the lack of evidence for effects on barren rate and lambing percentage in their study was likely due to exposure of ewes in all three categories of farms to SBV infection during pregnancy, including in the SBV not suspected farms used as the reference category for comparison of reproductive performance with that of SBV confirmed and suspected farms. Our post-outbreak analysis of farmer-maintained records was able to compare reproductive performance of early lambing flocks in the affected year with previous years, where data were available, and highlighted increased early embryonic losses in three early lambing flocks presenting as lower scanning percentages and higher ewe barren rates at pregnancy scanning in 2012/2013.

An important factor determining productivity and financial performance of lowland flocks is lamb rearing percentage (C. Phythian, Phillips, Wright, & Morgan, 2014). Although limited by small sample size, our results suggest there were greater lamb losses in SBV infected flocks that used oestrus synchronisation and artificial insemination (AI) as a breeding method than in those that naturally mated ewes in spontaneous oestrus. Rearing percentages for the two naturally mated flocks (B and D) were reduced by a factor of 0.91 and 0.88 times respectively compared to their baseline figures prior to the 2012 early lambing season, and approximately the same as the reduction in weaning rate (by a factor of 0.9, 10% fewer lambs) reported by Barrett et al. (2015) in SBV confirmed Irish flocks. Whereas, for three of the four flocks (A, E and F; not C) that artificially inseminated ewes at a synchronised oestrus, we were able to calculate a reduction in rearing percentage by a factor of 0.63 (37% fewer lambs reared); greater than that reported by Barrett et al. (2015).

Others have suggested flocks that synchronise oestrus to facilitate early breeding appear to be at higher risk of greater lamb losses due to SBV infection than those mating ewes naturally in spontaneous oestrus (Afonso et al., 2014; Simmons, 2012b). In this study, it seems plausible that in the three flocks that synchronised oestrus for AI to facilitate early breeding in July, a larger proportion of naïve ewes were at the critical stage of pregnancy for congenital malformations when they were first infected with SBV in summer 2012 than in the two flocks that mated ewes naturally in spontaneous oestrus in June 2012. It is not possible to determine whether the greater impact of SBV infection on reproductive performance in the three inseminated flocks was due to synchronisation of breeding for AI, to timing of early breeding in July compared to June, to differences in breeds of sheep, or to a combination of these or other factors.

Previous British studies have used a questionnaire survey, as was also used here, to quantitatively investigate farmers’ perceptions of the impacts of SBV disease outbreaks on flock welfare, financial performance, and their emotional well-being. This approach is not able to fully reflect the extent and complexity of farmers’ emotions and their perceptions of the impacts of SBV. However, the semi-structured interviews, carried out as part of this study, offered an opportunity to capture farmers’ responses to a series of open questions (see Supplementary Material S1 – Interview Questioning Guide) providing narrative descriptions of their lived experiences and perceptions of the impacts of SBV infection during the first outbreaks of disease in these early lambing flocks in 2012/2013. Farmers’ responses included descriptions of the distressing clinical signs observed around lambing time in ewes and lambs (see Supplementary Material S2 – Clip 1 – Liveborn Schmallenberg affected lamb with seizures; Clip 2 – Liveborn Schmallenberg affected blind ‘dummy’ lamb), the sometimes damaging interventions required to assist ewes (up to 92% [23/25] on one farm) to give birth to malformed lambs, and their perceptions of the negative impacts of the effects of SBV on: the welfare of ewes and lambs, the financial costs associated with increased ewe and lamb losses and reductions in lamb rearing percentage, veterinary and medicine costs including those associated with caesarian section, and subsequent treatment of ewes damaged during assisted delivery of malformed lambs, increased costs of rearing lambs, and effects on farmers’ emotional well-being. As has previously been reported in detail by C. J. Phythian and Glover (2019), and briefly here, farmers recalled their negative memories and emotions during that lambing period in terms such as: upset and touched by sheep losses, stressed, under pressure and tired, the difficulty, affect on mental health, depressing endings, and the ability to cope better normally at lambing time. However, those interviewed were experienced shepherds and some recalled experiencing both emotional ‘highs’ when things went well during lambing periods, and ‘lows’ when they did not e.g. during a previous abortion outbreak. Half of these farmers also recalled more positive emotions including feelings of resilience and ability to cope with this unexpected disease outbreak.

Farmers were asked to quantitatively score the severity of those impacts on a scale of 1 to 5 (1 = no impact, 5 = high impact) using the approach of Harris et al. (2014). All six farmers perceived negative impacts on animal welfare, flock financial performance, and their emotional well-being (Figure 3). Overall, the severity of impacts was perceived to be generally high, but self-appraised severity scores varied between farmers and, for five of the six farmers, between impacts. Variation in severity scores appeared not to be directly related to the proportion of lambs lost by flocks, as was also reported by Harris et al. (2014). There were indications from the qualitative interview data collected in this study that the severity of impacts perceived by farmers was variably influenced by multiple factors and effects of SBV infection including: increases in barren ewe rate; ewe and lamb mortality; reductions in lamb rearing percentage; births of living lambs with distressing clinical signs, particularly those with neurological abnormalities and requiring euthanasia; severe dystocia in a greater proportion of ewes requiring assistance to deliver malformed lambs; farmers’ experiencing both negative emotions of anxiety and depression associated with more intense feelings of tiredness and stress than during normal lambing periods, but also more positive emotions of resilience and ability to cope with the challenges and uncertainties of an unexpected and previously unknown disease outbreak. Harris et al. (2014) suggested uncertainty about the final outcomes of this novel disease, for which there were no effective control measures at the time of these first outbreaks, contributed to the severity of the impacts perceived by farmers. In our study, concerns and uncertainty were expressed about use of the SBV vaccine by two farmers who had decided not to vaccinate to protect their flocks. One farmer, recalled feeling uncertain about the safety, efficacy and use of the SBV vaccine at the time it was rapidly authorised and introduced to the market in May 2013; and another, who had experienced the lowest ewe and lamb losses of the six flocks during the first outbreak, and having decided not to vaccinate acknowledged some uncertainty about the risks of repeat SBV infection and future disease outbreaks.

Some farmers gave approximate estimates of the direct financial costs associated with reduced rearing percentages and lost income from lamb sales based on the value of slaughter lambs in 2012/2013. However, these estimates did not include losses due to increased ewe mortality and veterinary and medical costs, nor could they reflect the true value of lost genetic potential in ewes and lambs that died due to SBV. Farmer-perceived impacts on financial performance appeared high, irrespective of the number and proportion of lambs stillborn or that died in the neonatal period and irrespective of the reduction in rearing percentage. Other observed effects of SBV infection on reproductive performance, ewe dystocia and associated morbidity and poor milk production of ewes, are likely to have contributed to the high financial impacts perceived by farmers. In these high value pedigree and purebred flocks, some farmers appeared to be more upset by the long-term loss of valuable genetic material in the ewes and lambs that died, than by any short-term financial ‘hit’. Increased flock replacement costs associated with higher periparturient ewe mortality, and increased veterinary and medicine costs associated with dystocia and treatment of sick ewes, are also likely to have contributed to the perceived impact on financial performance, but are difficult to measure and were not assessed here, nor in previous British studies. Models have been developed to estimate the economic impact of SBV infection in a range of UK sheep production systems, but these have not included pedigree and purebred early lambing flocks deriving a proportion of income from sales of high value breeding sheep, and in which flock replacement costs are high (Alarcon et al., 2013).

Serological testing of these flocks following the first outbreaks of disease due to SBV infection in 2012/2013 indicated that within-flock seroprevalence was variable (34.9–93.6%) and natural immunity against SBV was likely to be incomplete (Mike Glover & Blake, 2013). These farmers were mostly risk averse to repeat SBV infection and future disease outbreaks and therefore it might seem surprising that only three of the six farmers decided to vaccinate their flocks prior to the next breeding season. The study design offered an opportunity to investigate farmers’ decisions on use of vaccine during the semi-structured interviews. Cost of vaccine did not appear to be a major factor influencing farmers’ decisions. Uncertainty about the safety of the newly developed vaccine and concerns about vaccinating sheep so close to the start of the breeding season were mentioned by one farmer who decided not to vaccinate. This farmer had felt reassured by veterinary advice that a large proportion of the flock was immune, blood testing for SBV antibodies in the serum of ewes had shown ~90% were seropositive, and risks of repeat infection were likely to be low; it seemed the farmer did not feel the likely benefit to be gained in terms of reduced risk of SBV infection outweighed the perceived risks associated with vaccination. Another farmer decided to delay the start of the subsequent breeding season by 1 month rather than vaccinate; an accepted management change to reduce the risk of disease outbreaks (Wernike & Beer, 2020). A third farmer did not offer a reason for not vaccinating; however, this flock lost the lowest proportion (4.1% [8/197]) of newborn lambs of the six flocks included in the current study, lost no ewes, and a low percentage of ewes (3.8% [4/105]) required lambing assistance to deliver affected lambs. This farmer perceived the severity of the impacts on flock welfare, financial performance and emotional well-being to be at the lowest level, and possibly did not perceive sufficient benefit in terms of disease risk reduction to justify the cost of whole flock SBV vaccination despite expressing some uncertainty about future risk. Larger studies have included more diverse samples of sheep farmers; these have found that between 13.3–13.7% of British farmers vaccinated with SBV vaccine in 2013, reducing to 1.6% in 2014 (Stokes, Baylis, & Duncan, 2016; Stokes et al., 2018). Stokes et al. (2018) suggested cost and previous experience of SBV were factors likely to influence vaccine uptake; following recrudescence of SBV in the UK in 2016/2017, a third of British farmers on SBV confirmed and suspected farms indicated they would consider vaccinating if the cost of vaccine was < £1, whereas fewer, around a quarter, would consider doing so on farms where SBV was not suspected; and < 10% of farmers would consider vaccinating if the cost of vaccine was £4–5. Uncertainty due to the lack of reported data on efficacy and duration of immunity when the first SBV vaccine was released, vaccine cost and previous experience of disease (Stokes et al., 2018), limited scientific knowledge about the disease, perceived isolated nature of the first disease outbreak and intermittent nature of future outbreaks, are likely to have influenced these and other farmers’ decisions on use of vaccination to prevent future outbreaks (Stavrou, Daly, Maddison, Gough, & Tarlinton, 2017).

Follow-up serological testing of homebred lambs born in 2014 indicated exposure of these study flocks to SBV was very low or unlikely in 2014 (Glover & Blake, unpublished observations), and furthermore, a freedom of disease study based on serological testing of lambs born in southern England between October 2014 and April 2015 indicated that circulation of SBV in the region was also very low, or unlikely, in 2015 (Stokes et al., 2016). The results of these two serological surveys, taken together with the absence of confirmed cases of SBV in the UK in 2015 (Anonymous, 2016), indicated there was likely to be an increasing proportion of naïve animals in flocks in the UK in 2016 (Stavrou et al., 2017). Subsequently, recirculation of SBV and recrudescence of disease were confirmed in the UK in autumn 2016 (Anonymous, 2016, 2017; Stokes et al., 2018) and again in 2021 (Anonymous, 2022), and it is expected this pattern of virus circulation and disease re-emergence will be repeated intermittently every 3–6 years (Anonymous, 2021; Stavrou et al., 2017).

Since the first outbreaks of disease were reported in 2012 the UK Government has funded passive surveillance for SBV in the UK, including free of charge PCR testing of the brain in lamb submissions fitting the case description for SBV and antibody testing of serum from ewes. However, looking forward passive surveillance is of limited value to sheep farmers such as those included in the current study, who in high risk years when SBV begins to circulate, might want to deploy preventive measures such as vaccinating part or all of their flocks (assuming vaccine is available) ahead of the breeding and peak midge vector seasons, or delaying the start of mating. Early warning systems would allow farmers to proactively deploy vaccine, or take other management decisions, to prevent disease outbreaks ahead of the breeding season (De Regge, 2016; Kirkland, 2015). A jointly funded cooperative initiative between the livestock industries and government (both national and state) established a national arbovirus monitoring system for arboviruses in Australia in 1993 to monitor annual fluctuations in viruses and vectors (Kirkland, 2004); this system could provide a model for a similar Europe-wide arbovirus (SBV and Bluetongue virus [BTV]) surveillance system (Stavrou et al., 2017).

### Study strengths and limitations

The small number of participating farmers, the nature of the farm visits and familiarity with the veterinarian, allowed us to collate good quality, detailed records, from almost all flocks, and to carry out the qualitative elements of this study. Whilst farmers likely to have good records were selected, farmers’ records were not perfect and we were not able to determine baseline figures for comparison for all reproductive parameters in all flocks. We also recognise that this study did not capture a saturation of farmers’ beliefs across the region in the qualitative research elements, nor did we capture the diversity of beliefs about the risk of future circulation of SBV and outbreaks of disease, and intentions to vaccinate to protect flocks. These flocks were purposively selected, they were high value pedigree and purebred flocks on farms in the southwest of England, and they lambed early between early November and late January 2012. As a result, these findings may not be generalisable to commercial flocks lambing later in the spring in other parts of the UK, and may not be directly comparable to those of previous British impact studies that were larger and included farms with a more diverse range of lambing dates and production systems, and estimated impacts at farm rather than flock level.

## Supplementary materials

Supplementary material S1 – Interview Questioning Guide

Supplementary material S2 – Video Clip 1: Liveborn Schmallenberg affected lamb with seizures; Video Clip 2: Liveborn Schmallenberg affected blind ‘dummy’ lamb

Supplementary material S3 – Table 1: Farmer recorded flock-level reproductive performance data for the production year including the November 2012 to January 2013 lambing period

Supplementary material S4 – Table 2: Farmer recorded flock-level data on the impacts of SBV on ewes and lambs, reproductive performance and survival around lambing time during the November 2012 to January 2013 lambing period

## Acknowledgements

We are grateful to the farmers who participated in this study for candidly sharing their experiences and allowing us access to their farm management records. We would also like to thank the two reviewers for their helpful comments on the manuscript.

## Author contributions

**Mike J. Glover:** Conceptualisation, Methodology, Funding acquisition, Investigation, Data curation (quantitative element), Formal analysis (quantitative element), Co-writing – First draft preparation, Co-writing – Review & Editing. **Neil Blake:** Conceptualisation, Methodology, Funding acquisition, Co-writing – Review & Editing. **Clare J. Phythian:** Conceptualisation, Methodology, Investigation, Data curation (qualitative element), Formal analysis (qualitative element), Co-writing – First draft preparation, Co-writing – Review & Editing.

### ORCID

Mike J. Glover: https://orcid.org/0000-0002-3454-0676

Neil Blake: https://orcid.org/0000-0002-1739-5120

Clare J Phythian: https://orcid.org/0000-0003-3140-4414

### Funding

We are grateful to MSD Animal Health, the South West Healthy Livestock Initiative, and the directors of Torch Farm and Equine Ltd. for their encouragement and financial support. The funders played no role in the study design, conduct, analysis, interpretation or reporting of these findings.

### Conflict of Interest

The authors declare no conflicts of interest.
